# A cross-sectional study of prevalence and predictors of risky sexual behavior among school-going adolescents in Mbarara municipality, Uganda

**DOI:** 10.4314/ahs.v23i3.14

**Published:** 2023-09

**Authors:** Michael U Anyanwu, Imelda Tamwesigire

**Affiliations:** Mbarara University of Science and Technology Faculty of Medicine, Department of Community Health

**Keywords:** Prevalence, risk factors, sexual behavior

## Abstract

**Background:**

Adolescents in Sub-Saharan Africa are at a greater risk of experiencing the adverse consequences of risky sexual behavior such as unwanted pregnancy and school drop-out than adolescents from other regions.

**Objectives:**

This study determined the prevalence and identified the demographic, school and social factors associated with risky sexual behavior among secondary school students in Uganda.

**Methods:**

This was a quantitative cross-sectional study conducted in 12 secondary schools in Mbarara Municipality, Uganda. A self-reported questionnaire was used to estimate the prevalence and predictors of risky sexual behavior among the students.

**Results:**

Out of the 910 students, 314 (34.6%) were sexually active and almost two in every ten adolescents had engaged in risky sexual behavior 171 (18.8%). About 27 (8.7%) had been pregnant or impregnated their sexual partner while 143 (45.6%) used condoms consistently. Risky sexual behavior was associated with age, gender of the student as well as alcohol consumption, smoking and substance use.

**Conclusion:**

Most of the participants were not sexually active, however, among the sexually active students, more than half engaged in risky sexual behavior. This finding suggests the need to introduce comprehensive sex education with a focus on safe sex practices in secondary schools in Uganda.

## Introduction

Adolescence is a period of exploration and experimentation often involving risky behaviors. Risky sexual behaviors among adolescents such as early sexual debut, unprotected sexual intercourse and multiple sex partners is a global health challenge resulting in new cases of human immunodeficiency virus (HIV) infections, sexually transmitted diseases (STDs), unwanted pregnancies and unsafe abortions 1-3. According to the World Health Organization (WHO), about one million cases of STDs are reported daily globally and adolescents account for approximately 50% of the cases 4. This has led to high rates of sexually transmitted diseases in adolescents compared to other age groups with over 30% of new HIV infections and majority of STDs occurring in this population each year 5, 6. Risky sexual behavior among adolescents has been associated with teenage pregnancy and abortion which is especially problematic in countries where there are restrictive abortion policies 7. About 5.6 million abortions that occur each year among adolescent girls globally, 3.9 million abortions are unsafe which have resulted to various complications affecting the mental and reproductive health of adolescents 7. Meanwhile, adolescents are less likely to have coping skills, information and necessary maturity required to protect themselves from unwanted pregnancy, STDs and HIV compared to adults 8.

Several factors such as personal, socio-cultural and economic factors predispose adolescents to risky sexual behavior 8–11. In Sub Saharan Africa (SSA), adolescents are at a greater risk of engaging in risky sexual behavior and experiencing the adverse effects compared to other regions of the world^7^, ^12^. This is largely due to restrictive abortion policies and lack of comprehensive sex education in the region 8, 13, 14. Adolescents in SSA generally initiate sex early, have multiple sexual partners, do not have access nor use contraceptives which increases the likelihood of contracting HIV, STDs, teenage pregnancies, unsafe abortions and school dropout 3, 7, 13, 15. A study conducted in Ethiopia showed that the prevalence of risky sexual behavior among adolescents was 19.6% 16 and another study conducted among secondary school students in Nigeria showed a prevalence of 24.1% 17. A study conducted among adolescents aged 10–19 years in Uganda reported that 62% of adolescents have ever had sex, out of which 11.4% had engaged in risky sexual behavior 18.

Uganda has a youthful population with about half of the citizens under the age of 25 years 19. It is also among the countries with a high prevalence of HIV/AIDS of 6.2% 20, and 25% teenage pregnancy 21. According to the Ugandan Demographic and Health Survey, 9% of the pregnancies are unwanted and 32% of sexually active unmarried women have an unmet need for family planning 21. Access to sexual and reproductive health (SRH) information still poses a challenge in Uganda. In 2016, the Ugandan Ministry of Gender, Labour and Social Development banned sex education in schools 22. Parents, religious leaders, and members of parliament feel that teaching sex education in schools will erode the culture and morality of the country and lead to unrestrained sexual activity among adolescents 23. Thus, with no SRH curriculum in secondary schools, some students seek SRH information from the media and their peers which result to access of explicit contents from the media and wrong SRH information 24, 25.

In 2018, the Ministry of Education and Sports released a national sexuality education framework which is a reference book for sex education within Uganda's schools 26. To reflect the traditional and religious values of the Ugandan society, the framework was based on abstinence rather than comprehensive sexuality education. Comprehensive sexuality education which is a curriculum based teaching provides scientifically accurate, realistic and non-judgmental information and equip students with knowledge, altitudes, skills and values needed to make appropriate and healthy choices in their sexual lives 27. It has been shown to be effective in improving SRH knowledge and safe sexual practices among adolescents 28–30.

No study has evaluated risky sexual behavior among secondary school students following the introduction of the national sexuality education framework as a guide to teaching of sex education and development of related materials in schools across Uganda. This study determined the prevalence of risky sexual behavior and identified the demographic, school and social factors associated with risky sexual behavior among school going adolescents in Uganda.

## Methods

### Study design and Setting

This was a cross sectional study conducted in secondary schools in Mbarara Municipality, Uganda between April and May, 2019. Mbarara Municipality is a town located in South Western part Uganda. It is the main municipal, administrative and commercial center of Mbarara District. Mbarara, is the largest city in the South-Western region, and located approximately 290 kilometers by road, southwest of Kampala, Uganda's capital city. Mbarara Municipality covers a total land area of about 51.47 square kilometers, and consists of six divisions.

### Participants and Procedure

There were 35 secondary schools with a total enrolment of 11,106 students in Mbarara Municipality (31, 32). Using multistage cluster sampling, 12 secondary schools were selected; two schools from each of Mbarara Municipality's six divisions. Each division was represented by one public and one private school. At each school, at least sixty students were randomly selected with minimum of ten students from each class using the class register. A total of 921 students were recruited for the study. A self-administered questionnaire was used to collect data. Research assistants that were trained, explained the objectives of the study to the students and assured them that all information was anonymous, no right or wrong answer and that answers will not affect their grades in any way. The questionnaire was given to all students that agreed to participate in the study and whom written consent was obtained.

### Ethical approval

The Research Ethics Committee of Mbarara University of Science and Technology approved the study. Authorization and written informed consent was obtained from the schools and students that participated in the study. The decision of students who were selected but not willing to participate in the study was respected and allowed to leave the classroom where the questionnaire was being administered. To ensure privacy and confidentiality of the participants, the desks and chairs in the classroom where the students completed the questionnaire were well spaced and unique codes were printed on the questionnaire with no name or personal identifiable data used in the questionnaire.

### Measures

Self-reported questionnaire was used to assess the sociodemographic characteristics such as age, gender, religion, residence and employment status of the students.

### Risky sexual behavior

Due to the sensitivity of the questions and the national guidelines on sexual and reproductive health information in schools, sexual behavior of the participants was assessed with questions on being sexually active, use of condoms and ever being pregnant. It was categorized into two: “No risky behavior” and “Risky Sexual behavior”. Respondent that answered “No” to being sexually active or “Yes” to being sexually active but “No” to her or their sexual partner ever being pregnant and “Yes” to consistent use of condoms was categorized as “No risky behavior”.

### Alcohol, smoking and substance use

Alcohol, smoking and substance use among the students was determined by the alcohol, smoking and substance involvement screening test (ASSIST), adapted using only question two (33, 34). It consists of 10 questions assessing alcohol, smoking and substance use in the past three months, with Never=0, Once/twice=2, Monthly=3, Weekly=4, and Daily=5. A score of “0” was classified as “no use” and 2 and above classified as “substance use”.

### Social support

Two questions assessed social support structures, “Is there advice and support provided by your family and friends for you?” and “Are there elders or community leaders that you can easily go to for support and advice? Social support was classified into “No social support” and “Have Social Support”. Any “Yes” to one of the questions was identified as having social support.

### Statistical analyses

After inspection of the questionnaires, the questions were coded, entered and analysed using STATA 12. The sexual behaviors and socio-demographic characteristics of the participants were summarized using frequencies and percentages. To identify the factors associated with risky sexual behavior, bivariate logistic regression was used. The variables that were significant in bivariate analysis were fitted into the multivariate model. Model goodness of fit test was checked using Hosmer-Lemeshow statistics (p = 0.35). All tests were two tailed and statistical significance level was set at p<0.05.

## Results

### Sample characteristics

Of the 921 study participants that was approached to take part in the study, 910 (98.8%) completed the questionnaires and 11 (1.2%) questionnaires were excluded due to missing sexual behavior information. The mean age and standard deviation of the students were 16.9 ± 2.2 with the majority aged below 18 years 480 (58.6%). A total of 486 (53.7%) were male students and most of the students were boarding students 681 (76.4%). Detailed baseline characteristics of the study participants are presented in Table 1.

**Table 1:** Demographics characteristics of the students

Variable	Total N = 910 (%)
Age Categories	
Below 18 years	480 (58.6)
18 years and above	339 (41.4)
Gender	
Female	419 (46.3)
Male	486 (53.7)
Class	
S1	128 14.1)
S2	126 (13.9)
S3	183 (20.2)
S4	199 (21.9)
S5	145 (16.0)
S6	126 (13.9)
Type of Student	
Boarding	681 (76.4)
Day	210 (23.6)
Type of School	
Private	501 (55.0)
Public	409 (45.0)
Mixed	788 (86.6)
Single	122 (13.4)
Residence	
Urban/Town	482 (57.2)
Sub-urban	360 (42.8)
Religion	
Catholic	330 (36.5)
Moslem	85 (9.4)
Protestant	175 (19.4)
Pentecostal	199 (22.0)
Other	114 (12.6)
Guardian/Caretaker	
Parents	791 (91.7)
Others	72 (8.3)
Orphan Status	
Both Parent Alive	731 (81.5)
One Parent Alive Orphan	136 (15.2)
Orphan	30 (3.3)
Part Time Job/Paid Work	
Yes	108 (12.7)
No	740 (87.3)

### Prevalence and predictors of risky sexual behavior

Out of the 910 students, 171 (18.8%) had engaged in risky sexual behavior. Overall, 314 (34.6%) of the students were sexually active from which 27 (8.7%) reported ever being pregnant or impregnating their sexual partners and 143 (45.6%) reported using condoms consistently. Unadjusted logistic regression analysis shows that age, gender, type of student, phone ownership and substance use were significant. After adjusting for potential confounders, age, gender and substance use were significant with students aged 18 years and above having the higher odds of engaging in risky sexual behaviors (AOR = 2.33, CI; 1.58–3.42). Also, male adolescents were 1.69 times more likely to practice risky sexual behavior than their female counterparts (CI; 1.15–2.50). Similarly, the odds of engaging in risky sexual behavior were 1.46 higher among students that drink, smoke and/or use substances compared to those that do not (CI; 1.01–2.12). The details are shown table 2.

**Table 2:** Predictors of risky sexual behavior

Variable	Risky sexual behavior		COR (95% CI)	P-value	AOR (95% CI)
	No n (%)	Yes n (%)			
Age Categories					
Below 18 years	418 (63.3)	62 (39.0)	1	<0.001	1
18 years and above	242 (36.7)	97 (61.0)	2.70 (1.89–3.86)		2.33 (1.58–3.42)***
Gender					
Female	364 (49.6)	55 (32.2)	1	<0.001	1
Male	370 (50.4)	116 (67.8)	2.07 (1.46–2.95)		1.69 (1.15–2.50) **
Type of Student					
Boarding	539 (74.6)	142 (84.5)	1.86 (1.19–2.92)	0.007	1.53 (0.95–2.46)
Day	184 (25.4)	26 (15.5)	1		1
Type of School					
Private	404 (54.7)	97 (56.7)	1.09 (0.78–1.52)	0.626	
Public	335 (45.3)	74 (43.3)	1		
Mixed	632 (85.5)	156 (91.2)	1.76 (0.99–3.11)	0.051	
Single	107 (14.5)	15 (8.8)	1		
Residence					
Urban/Town	391 (57.7)	73 (44.5)	1	0.612	
Sub-urban	287 (42.3)	91 (55.5)	1.09 (0.75–1.54)		
Religion					
Catholic	258 (35.2)	72 (42.4)	1.64 (1.02–2.62)		
Moslem	72 (9.8)	13 (7.7)	1.06 (0.52–2.15)	0.084	
Protestant	142 (19.4)	33 (19.4)	1.36 (0.79–2.35)		
Pentecostal	170 (23.2)	29 (17.1)	1		
Other	91 (12.4)	23 (13.5)	1.48 (0.81–2.70)		
Guardian/Caretaker					
Parents	647 (92.6)	144 (87.8)	1	0.05	
Others	52 (7.4)	20 (12.2)	1.73 (1.00–2.98)		
Part Time Job/Paid Work					
Yes	81 (11.7)	27 (17.1)	1.55 (0.96–2.49)	0.071	
No	609 (88.3)	131 (82.9)	1		
Phone Ownership					
No	357 (48.6)	58 (34.3)	1	0.001	1
Yes	377 (51.4)	111 (65.7)	1.81 (1.28–2.57)		1.23 (0.83–1.82)
Social Support					
No	119 (16.2)	35 (20.8)	1.38 (0.89–2.07)	0.154	
Yes	614 (83.8)	133 (79.2)	1		
Substance Use					
No	486 (66.5)	87 (51.5)	1	<0.001	1
Yes	245 (33.5)	82 (48.5)	1.87 (1.33–2.62)		1.46 (1.01–2.12) *

* p<0.05

** p<0.01

*** p<0.001

AOR=adjusted odds ratio; COR= crude odds ratio

## Discussion

In Uganda, several studies have been carried out on sexual behavior of various populations 18, 35–37. However, limited studies have been conducted among secondary school students after the introduction and use of the national sexuality education framework in the country. In our sample, the prevalence of risky sexual behavior was 18.8%. About 34.6% of the students reported being sexually active and majority of them was not using condoms consistently. Our study showed that older and male students were more likely to engage in risky sexual behaviors, and most importantly, alcohol consumption, smoking and substance use was significantly associated with risky sexual behavior.

Previous studies conducted before the introduction of the national sexuality education framework in Uganda reported different prevalence of risky sexual behaviors among adolescents. A study indicated that 18.1% of adolescents in rural setting in Uganda had ever had sex, out of which 18.5% had ever been pregnant 36. Another study showed that 7.6% of very young adolescents in primary schools had ever had sexual intercourse and 90% of the students were not using any protection 37. The estimated prevalence of risky sexual behavior in this study is however lower than that reported among adolescents aged 10–19 years old in a pastoralist post-conflict community in Uganda where 62% of the adolescents had ever had sex and 11.4% of all sexually active adolescents having engaged in risky sexual behaviors 18. In comparison to prior studies from other jurisdictions, the prevalence of risky sexual behavior in this study is similar to study findings in Ethiopia, 19.6% 16, and Nigeria, 24.1% 17 which have implemented the comprehensive sexuality education but lower than the prevalence reported in Tanzania, 41% 38 where there is no comprehensive sexuality education. Although, the estimated percentage of sexually active adolescents in this study tends to be similar to other studies, nonetheless, the percentage of inconsistent use of condoms is high among the students. Thus, it appears that whereas abstinence is important in this population, by itself it is not sufficient to prevent risky sexual behavior. This trend could be due to the abstinence only sexuality education in schools and barriers to obtaining contraceptives in Uganda.

The significant predictors of risky sexual behavior identified in this study were gender, age, alcohol consumption, smoking and substance use. Our study showed that male students were more likely to engage in risky sexual behavior compared to female students. This is consistent with previous studies 18, 36, although one study reported otherwise 39. This finding from our study could be due to youth programs which were targeted at the female gender. Also, socio-cultural values where males are supported to manifest their masculinity may encourage risky sexual behaviors among male students.

Older students aged 18 years and above were two times more likely to practice risky sexual behavior compared to younger adolescents. This is similar to other studies conducted in Uganda 21, 36. The age of 18 is the age of consent to sexual activity and this could explain why those aged 18 years and above are more likely to engage in risky sexual behavior. In consistent with other studies, adolescents that drink alcohol, smoke or use substances were more likely to engage in risky sexual behavior 18, 40–42.

Our study has limitations. Given the sensitive nature of this topic in Uganda, we could not include detailed risky sexual behaviors in the questionnaire. Also, all responses were self-reported and students may have given responses that were socially desirable. The scope of our study was limited to secondary school students in Mbarara Municipality; thus, the result of this study may not be generalized to all the adolescents in Uganda.

## Conclusion

Although majority of students reported not being sexually active, however, more than half of those that were sexually active engaged in risky sexual behavior. Male, older students and those that drinks, smokes or use substances were at a greater risk of engaging in risky sexual behavior. This study finding suggests that sexuality education need to be targeted at older and male adolescents, albeit including every adolescent in all youth and education programs. In addition, safe sex practices and other behaviors such as alcohol consumption, smoking and substance use that predispose adolescents to risky sexual behavior should be introduced in the sexuality education curriculum in schools.

## References

[1] Kazaura M, Masatu M (2009). sexual practices among unmarried adolescents in Tanzania. BMC Public Health.

[2] Kennedy S, Atwood K, Harris A, Taylor C, Gobeh M, Quaqua M (2012). HIV/STD risk behaviors amog in school adolescents in post-conflict Liberia. J Assoc Nurses AIDS Care.

[3] AnnDenise B, Jejeebhoy SJ, Iqbal S, Kathryn M (2001). sexual relations among young people in developing countries:evidence from WHO case studies.

[4] World Health Organisation (2018). Report on global sexually transmitted infection surveillance.

[5] WHO (2021). HIV and Youth.

[6] WHO (2021). Adolescent health epidemiology factsheet.

[7] Darroch J, Woog V, Bankole A, Ashford L (2016). Adding it up: Costs and benefitss of meeting the contraceptive needs of adolescents.

[8] Karl LD, Gabriele R, Marge B (2005). Sexually transmitted infections among adolescents: The need for adequate health services.

[9] Sacolo H, Chung M, Chu H (2013). High risk sexual behaviors for HIV among the in-school youth in Swaziland: A structural equation modeling approach. PLoS ONE.

[10] Steinberg L, Morris A (2001). Adolescent development. Annu Rev Psychol.

[11] Moreno M, Brockman L, Wasserheit J (2012). A pilot evaluation of older adolescents' sexual reference displays on Facebook. J Sex Res.

[12] Michael H, Sabrina B-K, Kizza L, Sara S, Philip L, Betsy P (2019). Depressive symptoms, sexual activity and substance use among adolescents in Kampala, Uganda. Afr Health Sci.

[13] United Nations (2014). Abortion Policies and Reproductive Health around the World.

[14] Louis O, Shittu R, Ajayi FA, Ameen A, Abdullateef G, Yusuf M (2019). High risk sexual behavior among adolescent senior school students in Nigeria. Afri Health Sci.

[15] Maly C, McClendon K, Baumgartner J (2017). Perceptions of adolescent pregnancy among teenage girls in Rakai, Uganda. Global Qualitat Nurs Res.

[16] Alem G, Teklewoini M (2019). Risky sexual behavior practice and associated factors among secondary and preparatory school students of Aksum town, northern Ethiopia, 2018. BMC Research Notes.

[17] Ajuwon AJ, Olaleye A, Faromoju B, Ladipo O (2006). Sexual behavior and experience of sexual coercion among secondary school students in three states in North Eastern Nigeria. BMC Public Health.

[18] Ssebunya RN, Matovu JKB, Makumbi FEea (2019). Factors associated with prior engagement in high-risk sexual behavior among adolescents (10–19years) in a pastoralist post-conflict community, Karamoja sub-region, North eastern Uganda. BMC Public Health.

[19] Uganda Bureau of Statistics, UBOS (2014). National population and housing census 2014, provisional results.

[20] Goverment of Uganda, Health Mo (2017). Uganda population-based HIV impact assessment (UPHIA 2016-2017).

[21] Uganda Bureau of Statistics, Calverton Ua (2012). Uganda demographic and health survey 2011.

[22] Cohen J, Tate T (2006). The Less the Know, the better: Abstinence-only HIV/Aids programs in Uganda. Reproductive health matters.

[23] Annah N (2016). The sex education debate has been blown out of proportion - UNFPA 2016.

[24] Miller A, Nalugya E, Gabolya C, Lagot S, Mulwanya R, Kiva J (2016). Ugandan adolescents' sources, interpretation and evaluation of sexual content in entertainment media programming. Sex Educ.

[25] Bull S, Levine D, Black S, Schmiege S, Santelli J (2012). social media-delivered sexual health intervention: a cluster randomized controlled trial. Am J Prev Med.

[26] Government of Uganda, Sports MoEa (2018). National sexuality education framework.

[27] UNESCO (2018). International technical guidance on sexuality education: an evidence-informed approach.

[28] Kemigisha E, Bruce K, Ivanova O (2019). Evaluation of a school based comprehensive sexuality education program among very young adolescents in rural Uganda. BMC Public Health.

[29] Fonner V, Armstrong K, Kennedy C, O'Reilly KR, Sweat M (2014). School based sex education and HIV prevention in low-and-middle-income countries: a systematic review and meta-analysis. PloS One.

[30] Kinsman J, Nakiyingi J, Kamali A, Carpenter L, Quigley M, Pool R (2001). Evaluation of a comprehensive school-based AIDS education programme in rural Masaka, Uganda. Health Educ Res.

[31] Ministry of Education and Sports, Department EPaP (2016). Education Abstract 2016.

[32] Uganda Bureau of Statistics (2017). The National Population and Housing Census 2014 – Area Specific Profile Series-Kampala Capital City Authority.

[33] World Health Organisation (2018). Alcohol, Smoking and Substance Involvement Screening Test.

[34] WHO ASSIST Working Group (2002). The Alcohol, Smoking and Substance Involvement Screening Test (ASSIST): development, reliability and feasibility. Addiction.

[35] Henry BM, Bakeera-Kitaka S, Lubega K, Snyder AS, LaRussa P, Pfeffer B (2019). Depressive symptoms, sexual activity, and substance use among adolescents in Kampala, Uganda. Afr Health Sci.

[36] Justine NB, Mary N, Tony S, Nathan I, David G, Wafaie WF (2020). Sexual bahaviors among adolescents in a rural setting in eastern Uganda: a cross-sectional study. Tropical Medicine and International Health.

[37] Kemigisha E, Bruce K, Nyakato VN (2018). Sexual health of very young adolescents in South Western Uganda: a cross-sectional assessment of sexual knowledge and bahavior. Reproductive Health.

[38] Mlunde LB, Poudel KC, Sunguya BF (2012). A call for parental monitoring to improve condom use among secondary school stuents in Dar es Salaam. Tanzania.

[39] Muluken D, Maereg W (2012). Predictors of consistent condom use among university students DrebreBerhan, Ethiopia. GJMEDPH.

[40] Geremew WW, Oyedunni A, Adebola R, Abraham SK (2019). Predictors of risky sexual behavior among pre-college students in Adama Town, Ethiopia. Pan Afr Med J.

[41] Hiwet ZF, Wondwossen L, Kidan AT (2015). Substance Abuse and predictors of risky sexual behavior among students in Axum University, Ethiopia. Journal of Addiction Research and Therapy.

[42] Kasirye R, Mutaawe R (2018). Sexual risks, substance abuse and protective factors among, Kampala Street and slum children. J ment Health Clin Psychol.

